# Exploring synthetic biology for the development of a sensor cell line for automated bioprocess control

**DOI:** 10.1038/s41598-022-06272-x

**Published:** 2022-02-10

**Authors:** Nikolas Zeh, Melina Bräuer, Nadja Raab, René Handrick, Kerstin Otte

**Affiliations:** Institute of Applied Biotechnology, University of Applied Sciences Biberach, Biberach, Germany

**Keywords:** Biotechnology, Expression systems, Molecular engineering

## Abstract

Unfavorable process conditions lead to adverse cultivation states, limited cell growth and thus hamper biotherapeutic protein production. Oxygen deficiency or hyperosmolality are among the most critical process conditions and therefore require continuous monitoring. We established a novel sensor CHO cell line with the ability to automatically sense and report unwanted process conditions by the expression of destabilized fluorescent proteins. To this end, an inducible real-time system to detect hypoxia by hypoxia response elements (HREs) of vascular endothelial growth factor (VEGF) origin reporting limitations by the expression of destabilized green fluorescent protein (GFP) was created. Additionally, we established a technique for observing hyperosmolality by exploiting osmotic response elements (OREs) for the expression of unstable blue fluorescent protein (BFP, FKBP-BFP), enabling the simultaneous automated supervision of two bioprocess parameters by using a dual sensor CHO cell line transfected with a multiplexable monitoring system. We finally also provided a fully automated in-line fluorescence microscopy-based setup to observe CHO cells and their response to varying culture conditions. In summary, we created the first CHO cell line, reporting unfavorable process parameters to the operator, and provided a novel and promising sensor technology accelerating the implementation of the process analytical technology (PAT) initiative by innovative solutions.

## Introduction

Optimized bioprocess conditions are essential to ensure strong cell growth, high productivity and good product quality during industrial biomanufacturing^1^. In particular, oxygen deficiency and hyperosmolality can be challenging parameters leading to unfavorable cellular behavior and significantly deteriorated performance^2,3^. In this context, a sufficient oxygen supply of at least 10 to 15% pO_2_^4^ is crucial to maintain critical energy metabolism contributing to physical cellular functions such as cell growth or autophagy^5–7^. Additionally, osmolality of more than 0.42 osm/kg^8^ can negatively influence a bioprocess, as hyperosmolality is reported to reduce growth and cellular viability and impair product quality, as increased salt concentrations may promote aggregation or change the glycosylation pattern of a biotherapeutic^9–11^. To avoid these drawbacks, continuous monitoring of critical process parameters is required by pO_2_ probes for oxygen and manual offline measurements for osmolality to detect and adjust deviations and sustain a successful bioprocess.. In the case of offline measurements requiring cell culture samples, the risk of contamination is also increased in combination with the need for additional hands-on time for periodical data points. Moreover, as adverse culture conditions are not autonomously communicated be the cell line, the targeted values for pO_2_ or osmolality are defined by the operator demanding excessive testing for each generated cell line to explore optimal conditions^12^. Therefore, the aim of the current study was to generate a novel sensor cell line that autonomously senses and reports unfavorable cultivation conditions during bioprocessing at the molecular signal level to the operator, with the scope to simplify measurements and improves insights into the modulation of intracellular physiological stages by in-line monitoring in real time. Especially for modelling approaches a cell line reporting its current state may be an important improvement^13^.

**Figure 1:**
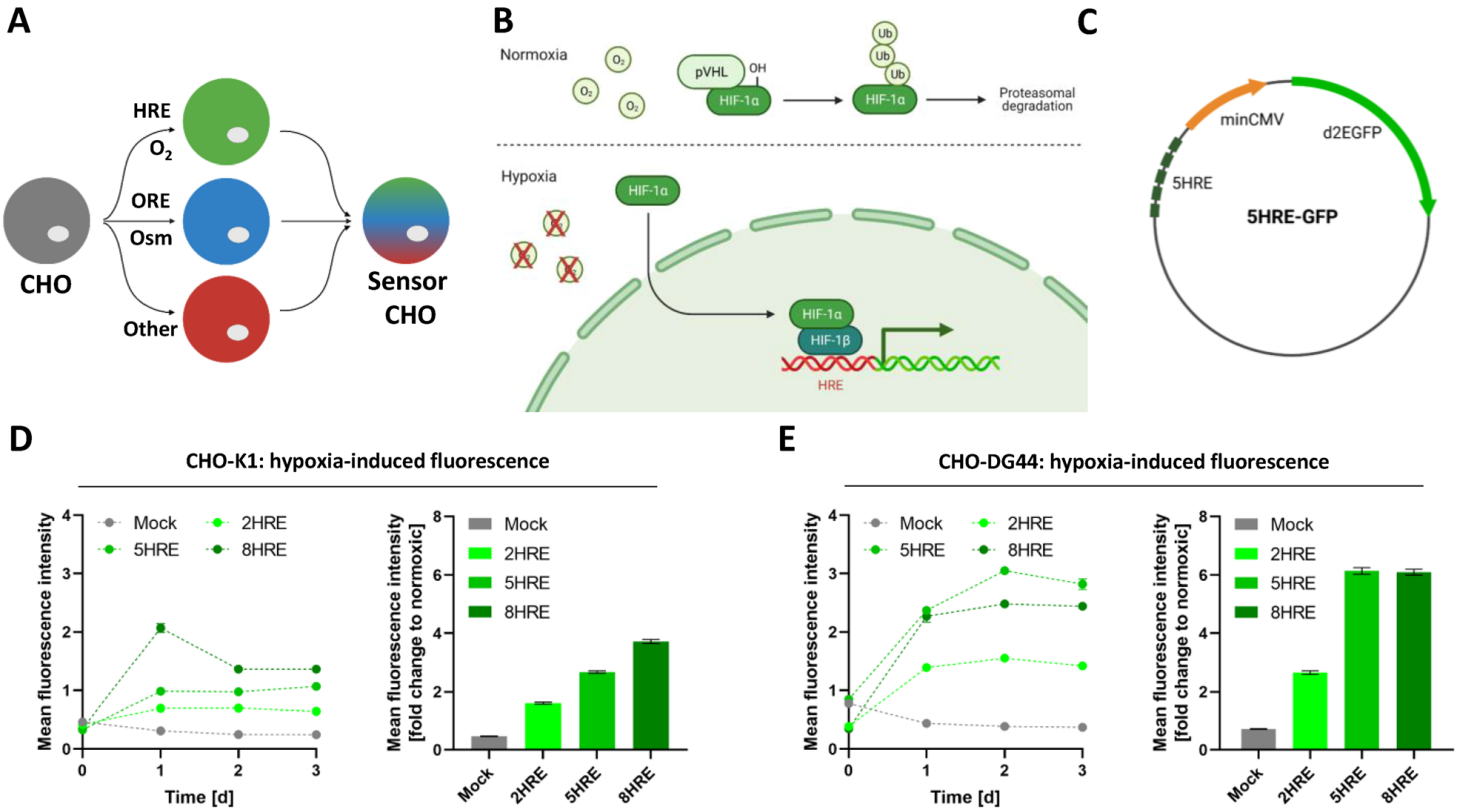
Establishment of a hypoxia-sensitive monitoring system. (**A**) Schematic overview of a CHO cell modified with different fluorescent expression vectors under the control of various response elements. (**B**) Hypoxia signaling pathway. (**C**) Schematic illustration of the hypoxia inducible d2GFP expression vector. (**D**) Flow cytometric analysis of shaken cultured CHO-K1 cells (left) and relative fold-change of mean fluorescence against the shaken cultured control (right). (**E**) Flow cytometric analysis of shaken cultured CHO-DG44 cells (left) and relative fold-change of mean fluorescence against the shaken cultured control (right).

In nature, mammalian cells exploit the use of response elements to sense and react to hostile environmental conditions^14–16^. Oxygen deficiency, for example, leads to the stabilization of hypoxia-inducing factor 1α (HIF-1α), which dimerizes with its cofactor HIF-1β to interact with hypoxia response elements (HREs) and activates the expression of protective genes such as erythropoietin (EPO) or vascular endothelial growth factor A (VEGF-A) (Fig. 1B) to perpetuate cellular physiological functions^17–20^.

A comparable mechanism preventing cells from receiving damage by external factors represents the response to hyperosmolality^21,22^. High salt concentrations lead to enhanced mRNA stabilization of the tonicity response element binding protein (TonEBP), better known as nuclear factor of activated T-cells 5 (NFAT5)^23^. As a consequence, enriched amounts of NFAT5 protein are phosphorylated and translocate into the nucleus for binding to osmotic response elements (OREs) of osmoprotective genes such as aldose reductase (AR) or secretin (SCT) and secretin receptor (SCTR)^22–25^. In this context, NFAT5 features comparable competencies to HIF-1α, protecting cells from external influences and maintaining cellular functions by interacting with short, repetitive DNA sequences to coordinate target gene transcription.

In the current study, we aimed to generate a novel sensor cell line, reporting unfavorable cultivation conditions during bioprocessing by exploiting response elements for hypoxia and hyperosmolality inspired by nature. Specifically, we established an in-line oxygen reporter system in CHO cells by the expression of destabilized GFP (d2GFP) under the control of HREs of VEGF origin. Additionally, we showed the functionality of OREs of AR origin by the expression of unstable BFP (FKBP-BFP) to enable simultaneous detection of multiple process parameters. Finally, we were able to demonstrate independent functionality of the introduced monitoring systems and to set up an in-line automatically functional fluorescence microscope-based method to enable observation of CHO cells close to real time.

## Results

### Development of a genetic sensing system for oxygen deprivation

To simplify the measurement of unfavorable bioprocess conditions, we aimed to enable the CHO cells themselves to directly sense and report a changing environment within the cell culture vessel. Using a synthetic biology approach, we envisaged genetic response elements (REs) to drive destabilized fluorescent proteins. Different cellular fluorescence would then directly indicate changing bioprocess conditions (Fig. 1A). Since the available oxygen concentration is one of the main bioprocess conditions constantly monitored, we turned to the genetic control of oxygen sensing in mammalian cells. In this study, normoxia is defined as oxygen concentrations of 40% to 60% pO_2_, while hypoxia describes pO_2_ values of < 15%. The hypoxia response pathway is mainly controlled by hypoxia-inducing factor-1α (HIF-1α), which is degraded during normoxia. Under oxygen deprivation conditions, HIF-1α is stabilized and binds together with HIF-1β to hypoxia response elements (HREs) to activate the expression of numerous genes (Fig. 1B). Previously, we showed the expression of relevant hypoxia response transcription factors in CHO cells^4^.

To establish a synthetic genetic sensing system for oxygen limitation in mammalian production cells, vectors were constructed linking a minimal CMV promoter to ensure expression of the fluorescent protein only under specified conditions to HREs driving the expression of destabilized GFP (d2GFP) (Fig. 1C). After stable transfection of expression vectors carrying two, five or eight canonical repetitive HREs into CHO-K1 and CHO-DG44 cells, the functionality of the synthetic oxygen sensing system was evaluated by culturing cells under shaken or oxygen-limiting static conditions. Engineered CHO-K1 cells showed an increased fluorescence signal after one day of static cultivation, while mock cells lacking HREs in front of the minimal CMV maintained GFP fluorescence at a low basal level of 0.3 mean fluorescence (Fig. 1D). Furthermore, a correlation of GFP expression and the number of HRE repeats was observed, with the strongest signal for 8 HREs showing 2.1 mean fluorescence (Fig. 1D). When comparing statically (hypoxic) cultured CHO-K1 cells with their shaken (normoxic) cultured replica, an equal d2GFP signal was detected for CHO-K1-mock cells, but amplified expression of d2GFP (2- to 4-fold) was observed for the HRE construct harboring CHO cells (Fig. 1D). Similar observations were obtained for CHO-DG44 cells transfected with a mock- or HRE-encoding vector. While the mock cell line showed no inducible d2GFP expression under oxygen-limiting conditions (0.5 mean fluorescence), HREs led to a strong increase in the mean fluorescence signal of 1.6 to 3.1 (Fig. 1E). Compared to the shaken cultivated mock cell line, CHO-DG44 cells exhibited an even stronger up to 6-fold increase in d2GFP expression and outperformed CHO-K1 cell lines (up to 4-fold) grown under hypoxic conditions (Fig. 1D,E). In contrast to CHO-K1 cells showing the best inducibility of d2GFP expression with 8 HREs, the construct with 5 HREs led to the best results in CHO-DG44 cells (Fig. 1D,E). Additionally, basal expression of d2GFP under the leakage of minimal CMV was very low, with less than 0.2 mean fluorescence.

### Expanding the sensing system to hyperosmolality

**Figure 2:**
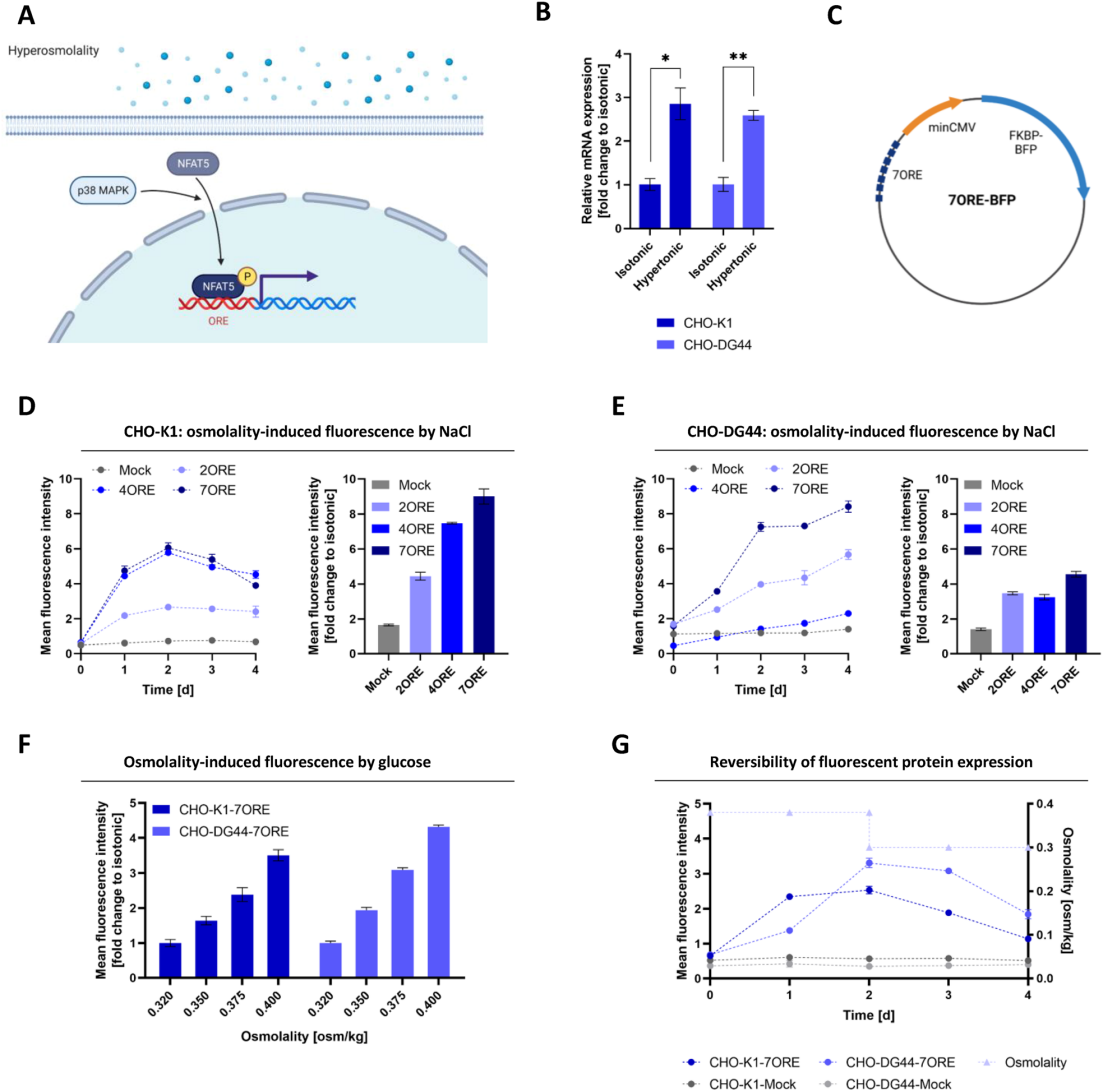
Development of a hyperosmolality-sensitive expression system. (**A**) Hyperosmolality signaling pathway. (**B**) qPCR of NFAT5 expression in CHO-K1 and CHO-K1-DG44 cells under isotonic and hyperosmotic conditions. (**C**) Schematic illustration of the hyperosmolality inducible FKBP-BFP expression vector. (**D**) Flow cytometric analysis of NaCl-induced hyperosmotic cultured CHO-K1 cells (left) and relative fold-change of mean fluorescence against the isotonic cultured control (right). (**E**) Flow cytometric analysis of NaCl-induced hyperosmotic cultured CHO-DG44 cells (left) and relative fold-change of mean fluorescence against the isotonic cultured control (right). (**F**) Flow cytometric analysis of glucose-induced hyperosmotic cultured CHO-K1 and -DG44 cells with relative fold-change of mean fluorescence against the isotonic cultured control. (**G**) Flow cytometric analysis of hyperosmotic cultured CHO-K1 and -DG44 cells with restoration of isotonic conditions after 2 days. [n = 3 replicates, Mean ± SD; * = *p* < 0.05; ** = *p* < 0.01].

After developing a genetic sensor system for unfavorable bioprocess conditions, as exemplified by oxygen limiting conditions, we aimed to establish a synthetic multiplex sensor system. For bioprocess monitoring, osmolality is an important parameter; therefore, we searched for a genetic system for sensing and reporting. In this study, isotonic conditions are defined between 0.30 to 0.33 osm/kg and hyperosmolality is characterized by > 0.40 osm/kg. In mammalian cells, osmolality is mainly sensed by phosphorylation states and expression levels of nuclear factor of activated T-cells 5 (NFAT5)^24,25^. During hyperosmolality, NFAT5 mRNA is stabilized, leading to higher amounts of NFAT5 protein, which enters the nucleus after phosphorylation as a transcription factor and activates gene expression by binding to osmolality response elements (OREs) (Fig. 2A)^23^. To assess the potential of this genetic system for CHO cells, the expression levels of NFAT5 under isotonic (0.3 osm/kg) and hyperosmotic (0.4 osm/kg) conditions were analyzed. For both CHO cell derivatives K1 and DG44, NFAT5 expression was confirmed, even showing a 2.9-fold (CHO-K1) and 2.6-fold (CHO-DG44) elevation of NFAT5 transcripts at hyperosmolality (Fig. 2B).

Since both the presence and functionality of this crucial factor for sensing osmolality were verified, vectors were constructed following the same principle as before by linking a minimal CMV promoter to OREs of AR origin driving the expression of a destabilized BFP variant (FKBP-BFP), including zeocin resistance (Fig. 2C).

After stable transfection of CHO-K1 cells with vectors harboring various numbers of OREs, hyperosmotic culture conditions (0.45 osm/kg) were triggered by NaCl addition. While CHO-K1 mock cells did not induce FKBP-BFP expression and only showed a low basal mean fluorescence of 0.6 (Fig. 2D), introduction of OREs led to a strong fluorescence induction of up to 6.1 mean fluorescence, which was correlated with the ORE number (Fig. 2D). Especially in comparison to isotonic cultured cell lines, an up to 9-fold increased FKBP-BFP signal was obtained for the best construct harboring 7OREs (Fig. 2D). CHO-DG44 cells transfected with either the mock or ORE vectors revealed comparable results with slightly higher basal expression for mock but also showed up to 8.4 mean fluorescence at hyperosmolality for the 7ORE-containing construct (Fig. 2E). In addition, OREs in CHO-DG44 cells led to a 4- to 5-fold pronounced FKBP-BFP expression during cultivation in hyperosmotic (0.45 osm/kg) versus isotonic (0.32 osm/kg) conditions (Fig. 2E). As osmolality in fermentations is influenced not only by Na^+^ ions of pH correction agents but also by supplemental feeds, the impact of glucose on the synthetic sensor system was assessed. For both CHO-K1-7ORE and CHO-DG44-7ORE cell lines, a comparable induction with a linear correlation of FKBP-BFP expression to the applied osmolality was detected (Fig. 2F). Finally, the reversibility of FKBP-BFP expression was demonstrated by reversing hyperosmotic conditions to isotonic conditions and nearly restoring the basal expression values of noninduced CHO-K1-7ORE or DG44-7ORE cells. Therefore, the established osmolality sensing system was validated to be suitable for monitoring osmolality during fermentations (Fig. 2G).

### Multiplex detection of bioprocess parameters by CHO sensor cell line

**Figure 3:**
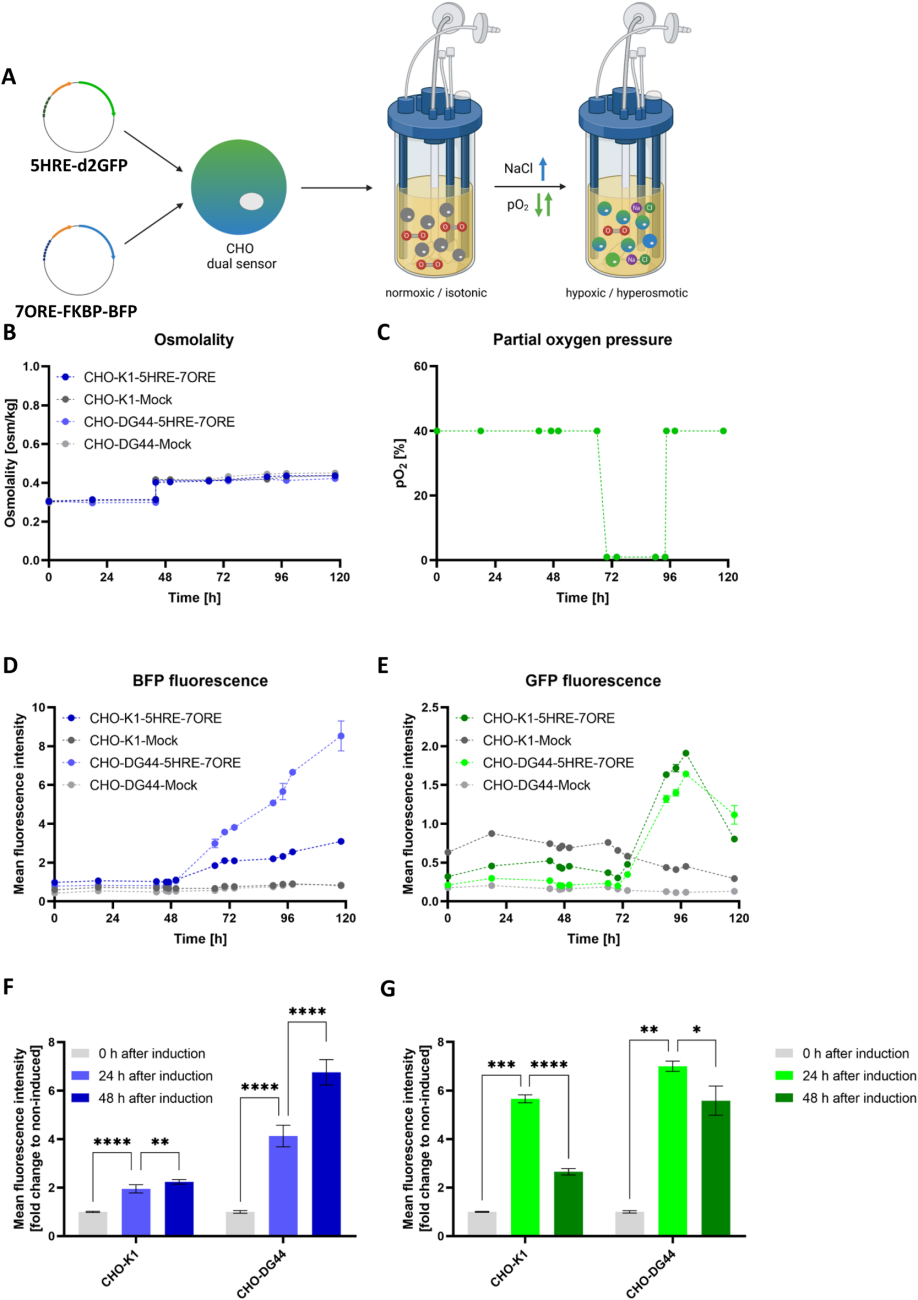
Batch fermentation of dual sensor cell lines under hypoxic and hyperosmotic conditions. (**A**) Schematic experimental setup. (**B**) Osmolality during batch fermentation. (**C**) pO_2_ concentration during batch fermentation. (**D **,**E**) Flow cytometric analysis of CHO-K1 and -DG44 cells during batch fermentation expressing FKBP-BFP (D) and d2GFP (**E**). (**F** ‚**G**) Relative fold-change of FKBP-BFP (**F**) or d2GFP (**G**) mean fluorescence against the mock control cell line during batch fermentation.

Following the establishment of a synthetic sensor system to monitor oxygen limitation or hyperosmolality by the exploitation of genetic response elements, we aimed to multiplex the inducible reporter systems to sense both parameters simultaneously. Therefore, stable multiplex sensor CHO-K1 and CHO-DG44 cell lines were created expressing both the 5HRE-d2GFP and 7ORE-FKBP-BFP expression vectors (Fig. 3A) and subsequently functionally validated in batch fermentation (Fig. 3A). Growth profiles and viabilities of all batch fermentations are shown in Supplemental Figure 1. After 48 h, the osmolality was increased from 0.31 osm/kg to 0.41 osm/kg to trigger FKBP-BFP expression (Fig. 3B), followed by a reduction in the pO_2_ set point to 1% after 72 h to trigger the HRE-dependent d2GFP signal (Fig. 3C). For both the CHO-K1 and CHO-DG44 sensor cell lines, increased FKBP-BFP expression was measured when osmolality was increased, although CHO-DG44 cells (8.5 mean fluorescence) revealed a stronger FKBP-BFP signal than CHO-K1 cells (3.1 mean fluorescence) (Fig. 3D). In comparison, CHO-K1 and CHO-DG44 control cell lines missing response elements for hypoxia or osmolality maintained low basal expression throughout the whole batch fermentation for both conditions (Fig. 3D,E). Similar to the induction of FKBP-BFP by hyperosmolality, d2GFP expression was also triggered (CHO-K1: 1.9 mean fluorescence; CHO-DG44: 1.6 mean fluorescence) after establishing oxygen limitations, demonstrating the functionality and the possibility to multiplex response elements to monitor multiple cultivation conditions simultaneously (Fig. 3E). Finally, the reversibility of the system was analyzed by restoring 40% pO_2_ after 96 h, which inhibited the expression of d2GFP and reduced the fluorescence signal for CHO-K1 and –DG44 cell lines to approximately 0.8 and 1.1 mean fluorescence, respectively (Fig. 3C,E). Overall, CHO-DG44 cells generally display a greater inducibility when comparing the FKBP-BFP or d2GFP signal of 5HRE- and 7ORE-harboring expression cell lines to their equally cultivated controls, which resulted in a significant up to ~ 6-fold amplification for both FKBP-BFP and d2GFP after induction (Fig. 3F,G).

### Automated real-time monitoring of a CHO multiplex sensor cell line

**Figure 4:**
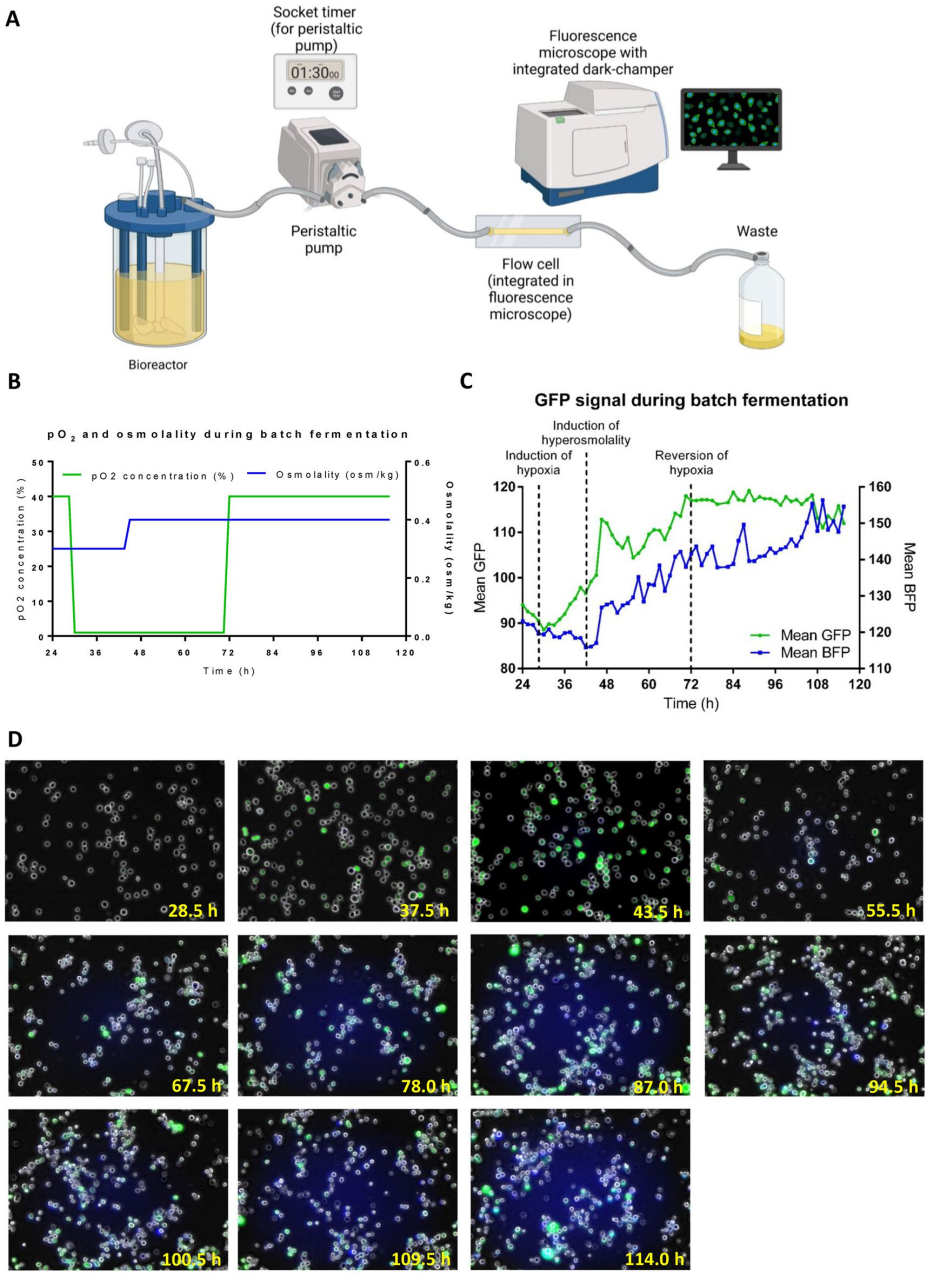
Automated real-time monitoring of a CHO multiplex sensor cell line during batch fermentation. (**A**) Schematic experimental setup. (**B**) Osmolality and pO_2_ concentration during batch fermentation. (**C**) d2GFP and FKBP-BFP signals of CHO-DG44 cells during batch fermentation recorded by image cytometry at 90 min intervals. (**D**) Representative fluorescence microscopy images of CHO-DG44 cells during batch fermentation recorded at 90 min intervals.

After demonstrating the functionality of a multiplex sensor CHO cell line to monitor hyperosmolality and hypoxia simultaneously during batch fermentation, we created a fully automated in-line system to observe the cellular state close to real-time. Therefore, we performed batch fermentation with the above established CHO-DG44 multiplex sensor cell line and used a peristaltic pump controlled by a socket timer to automatically flush the cell culture suspension in a 90 min interval through a flow cell implemented in a fluorescence microscope (Fig. 4A). In a synchronized manner, fluorescence microscopy provided five qualitative images distributed over the flow cell and quantitative cytoimaging results for each time point. For the induction of hypoxia or hyperosmolality, the previously established conditions were applied with a pO_2_ switch from 40 to 1% and an increase in osmolality from 0.3 to 0.4 osm/kg (Fig. 4B).

A decrease in the d2GFP signal was observed to 89 mean fluorescence until the pO_2_ set point was decreased to 1% and hypoxia was established (Fig. 4C). Subsequently, the d2GFP mean fluorescence increased continuously until 72 h and restoration of 40% pO_2_, where fluorescence started to stagnate at approximately 117 mean fluorescence (Fig. 4C,D). Notably, for d2GFP expression, the measured FKBP-BFP signal also decreased at the beginning of batch fermentation to 115 mean fluorescence until osmolality was adjusted to 0.4 osm/kg and hyperosmolality was established (Fig. 4C). Thereafter, a comparable nearly linear increase in FKBP-BFP expression was detected, reaching up to 156 mean fluorescence toward the end of the process (Fig. 4C,D). When the oxygen concentration was revered to 40% pO_2_ and normoxia was restored, the d2GFP signal stopped to increase but was not decreasing until a slight decrease of the d2GFP expression was visible during the end of the fermentation. Interestingly, the recorded fluorescence images revealed a heterogenic population of strong- and weak-expressing cells for d2GFP and FKBP-BPF during the different states of induction (Fig. 4D). In this context, green, blue and cyan cells were recorded, indicating cell stress by hypoxia, hyperosmolality or both (Fig. 4D). Finally, fluorescent pictures indicated an attachment of cells in the flow channel between 48 and 72 h, which revealed d2GFP expression due to hypoxic conditions inside chamber and explained the slight decrease of the d2GFP expression after normoxia was restored.

## Discussion

The production of complex biotherapeutics is mainly performed in CHO cells using bioprocesses in large-scale industrial fermenters^26^. To obtain ideal processes, including strong cell growth, high product titer and good product quality, constant monitoring of critical parameters and their maintenance in tight ranges is mandatory^1^.

Our work aimed to establish a novel and innovative solution by generating a self-reporting sensor cell line responding to unfavorable process conditions through the expression of unstable fluorescent proteins. While HREs of VEGF origin in CHO cells were shown to elevate the expression of recombinant proteins during oxygen limitations, HREs can also be utilized to control the expression of destabilized GFP (d2GFP) as a detectable marker for hypoxia. Our results indicate the functionality of HREs in both CHO-K1 and CHO-DG44 cells, with a stronger efficiency of HREs in CHO-DG44 cells. This finding was in concordance with our previous results revealing reduced expression and stabilization of the crucial hypoxia reporter HIF-1α in CHO-K1 cells in the absence of oxygen^4^. However, inducibility of d2GFP was sufficient for both cell lines to enable monitoring of oxygen limitations by expressing the fluorescent reporter molecule.

To multiplex the detection of several important bioprocess parameters, we also worked on the introduction of OREs to generate hyperosmolality-sensitive CHO cells.. Although overexpression of NFAT5 under hyperosmolality in CHO cells was shown before^27^, OREs have not yet been utilized and require detailed testing of several arrangements and conditions to unveil the most potent setup for reporting hyperosmolality by inducible FKBP-BFP expression. We were able to identify NFAT5 upregulation during hyperosmolality and achieved a nearly linear correlation of FKBP-BFP expression with both the number of ORE repeats and osmolality. Furthermore, our findings indicate the most promising inducibility by employing 7ORE repeats with comparable results in CHO-K1 and -DG44.

As proof of concept, an automated fluorescence microscopy-based setup was developed, enabling the observation and quantification of both fluorescence proteins in a continuous, close to real-time manner. Since we successfully introduced two independent systems for bioprocess monitoring, the possibility of multiplexing up to four bioprocess conditions with nonoverlapping fluorophores seems feasible when establishing suitable response elements^28^. In particular, the destabilization domain FKBP may be an interesting tool to generate further unstable fluorescent proteins by N-terminal fusion to fluorescent proteins, as applied for BFP^29^. Therefore, available fluorescent proteins such as RFP or miRFP670 could be modified to generate unstable, noninterfering versions for monitoring additional bioprocess conditions, as thus far, no efficient red or near infrared fluorescent proteins are available^29–31^. In this context, it is also important to develop further green or blue fluorescent proteins with even shorter half-life times than the 2 h of the utilized fluorescent proteins to generate a more precise and functional monitoring system. Although in our experiments restoration of normoxic or isotonic conditions led to decreased fluorescence expression, the basal mean fluorescence signal of noninduced CHO cells was not reached, and faster turnover of the mean fluorescence signal would be necessary for industrial applications. However, over the past years, a broad range of novel fluorescent proteins have been developed for the continuously growing requirements of novel diagnostic or imaging applications^32–34^.

As our study mainly served as a first of its kind proof of concept study, serval points have to be addressed to enable its industrial use like the monitoring speed, reporting strength and precision. Thereby the sensitivity of the system has to be optimized to enable the detection of the reporter molecule as soon as a limitation occurs and as fast as possible before adverse effects affect the cell culture ideally within 2–3 h. Another limitation represents the half-life time of the fluorescent dyes which has to be shorter than 2 h to reach a higher precision. In addition, the expression of free fluorescent proteins could be problematic for downstream processing (DSP) and may therefore represent an issue for regulatory authorities. Thus, alternative reporting systems are required in the future. Here, fluorophores linked to marker molecules of natural side products of CHO cells as exosomes may represent a potential alternative mediator of unfavorable conditions with less interference in DSP^35^.

As mentioned before, a triple or even quadruple sensor cell line should be feasible when suitable response elements are identified. Glucose deprivation is communicated in nature by the activation of glyconeogenesis, which is conducted by the upregulation of phosphoenolpyruvate carboxykinase (PEPCK) or pyruvate carboxylase (PC) via interaction of the cAMP response element binding protein (CREB) with cAMP response elements (CREs) in front of crucial glyconeogenic genes^36–39^. Adaptation of CREs and their linkage to an additional fluorescent protein may enable CHO cells to report starvation and lead to novel feeding regimes exploiting response elements as tools for automated sensing systems.

## Methods

### Cell culture

Suspension-adapted CHO-DG44 (A1100001, Thermo Fisher, Darmstadt, Germany) and CHO-K1 (CCL-61TM, ATCC) cells were cultured in chemically defined HyClone™ SFM4CHO™ medium (Cytiva, Marlborough, MA, USA) supplemented with 10 g/L glucose (Roth, Karlsruhe, Germany) and 4 mM l-glutamine (Lonza, Basel, Switzerland) at 37 °C, 5% CO_2_, 85% humidity, and 140 rpm (25 mm orbit) in shake flasks (Corning Inc, New York, USA) using a Kuhner incubate shaker (Adolf Kühner AG, Basel, Swiss). Subculturing was performed every 3–4 days to a viable cell density (VCD) of 0.5 × 10^6^ cells/mL. VCD and viability were determined by trypan blue exclusion using CEDEX XS Cell Analyzer (Roche Diagnostics, Mannheim, Germany). CHO cells were transfected with 15 µg vector using the NEON® transfection system (Thermo Fisher Scientific, Waltham, MA, USA). Selection of polyclonal cell lines stably expressing derivatives of the pEF-myc-cyto-mCMV-d2GFP vector (Addgene, Watertown, MA, USA) was performed using 500 µg/mL G418 sulfate (Genaxxon Bioscience, Ulm, Germany) and/or 400 µg/mL Zeocin (InvivoGen, Toulouse, France). All utilized vectors are listed in Table 1. To induce fluorescent protein expression, CHO cells were seeded with a VCD of 0.5 × 10^6^ cells/mL and grown statically for several days at undefined hypoxic conditions (37 °C, 5% CO_2_, and 85% humidity) in a Forma™ Steri-Cycle™ incubator (Thermo Fisher Scientific, Waltham, MA, USA) where a static cultivation and sedimentation of the cells was sufficient to generate hypoxic conditions. In addition, a shaken control batch was inoculated with a VCD of 0.5 × 10^6^ cells/mL to meme normoxic conditions. To induce fluorescent protein expression by OREs, hyperosmotic conditions (up to 0.45 osm/kg) were created by addition of sodium chloride (NaCl) or glucose and compared to an isotonic (0.3 osm/kg) cultured control. To analyze the reversibility of the fluorescent protein expression after hyperosmotic induction of 0.4 osm/kg, the cells were centrifuged and the media was changed to isotonic media with 0.3 osm/kg. The osmolality of culture supernatants was measured using OSMOMAT 030 (Gonotec, Berlin, Germany).

**Table 1:** Generated vector constructs for the analysis of HRE and ORE functionality.

Name (vector/cell line)	Vector backbone	Fluorophore	Response element	RE repetitions	Selectable marker
Mock-GFP	pEF-myc-cyto-mCMV	d2GFP	–	–	Neo
2HRE-GFP	pEF-myc-cyto-mCMV	d2GFP	VEGF-HRE	2	Neo
5HRE-GFP	pEF-myc-cyto-mCMV	d2GFP	VEGF-HRE	5	Neo
8HRE-GFP	pEF-myc-cyto-mCMV	d2GFP	VEGF-HRE	8	Neo
Mock-BFP	pEF-myc-cyto-mCMV	FKBP-BFP	–	–	Zeo
2ORE-BFP	pEF-myc-cyto-mCMV	FKBP-BFP	AR-ORE	2	Zeo
4ORE-BFP	pEF-myc-cyto-mCMV	FKBP-BFP	AR-ORE	4	Zeo
7ORE-BFP	pEF-myc-cyto-mCMV	FKBP-BFP	AR-ORE	7	Zeo

### Batch fermentation

For cultivation at defined O_2_ concentrations and hyperosmotic conditions, CHO cells were inoculated at a VCD of 0.7 × 10^6^ cells/mL in a volume of 1 L using 2 L stirred tank benchtop bioreactors (Sartorius, Göttingen, Germany). Process conditions were controlled at a stirring speed of 100 rpm, pH of 7.15, and temperature of 37 °C. The O_2_ set point was adjusted at different time points by gassing with N_2_ to reach a pO_2_ of 1%. To restore normoxia (pO_2_ = 40%) the gas mix was adjusted by the fermentation head-unit and air was used to enhance the desired pO_2_. If air was not sufficient to provide the desired O_2_ concentration, the fermentation head-unit mixed pure O_2_ into the gas mix. Osmolality was increased by the addition of a 3 M NaCl solution with a peristaltic pump and the volume of added NaCl was observed by a scale. We aimed to provide an increased osmolality of around 0.4 osm/kg and confirmed a final osmolality after NaCl addition of 0.41 osm/kg by external measurement of the cell culture suspension.

### Molecular biology

The starting plasmid 5HRE/GFP was a gift from Martin Brown & Thomas Foster (Addgene plasmid # 46926)^40^. Constructs were cloned using HRE sequences of VEGF and ORE sequences of AR published by Javan et al. 2017^16^ and Ferraris et al. 1999^41^, respectively. The oligos used are listed in Table 2. Prior to cloning, oligonucleotides were annealed by heating to 95 °C followed by slow cooling to 25 °C, phosphorylation using T4 PNK (New England Biolabs, Ipswich, MA, USA), and ligation by T4 ligase (New England Biolabs, Ipswich, MA, USA). After removing the 5HREs by digestion with *Xho*I and *Bgl*II (both New England Biolabs, Ipswich, MA, USA), the randomly ligated double-stranded oligonucleotides were cloned into the pEF-myc-cyto-mCMV-d2GFP vector. The origin of the destabilized blue fluorescent protein variant FKBP-mTagBFP2 and zeocin resistance for the exchange of fluorescent protein and selectable markers were the vectors pAW63.YY1.FKBP.knock-in. BFP (Addgene plasmid #104371)^29^ and pcDNA3.1/Zeo(+) (Thermo Fisher Scientific, Waltham, MA, USA), respectively. FKBP-mTagBFP2 and zeocin resistance were amplified via PCR using the following primers: FKBP-BFP Fwd (5-ATATCCATGGGTGCCCCTTCGACGGTTGTA-3); FKBP-BFP Rev (5-ATATTCTAGACTTCCCGGGTCGAGAAGGTC-3); Zeo Fwd (5′-ATATTCTAGACCCGTTTAAACCCGCTGA-3′); and Zeo Rev (5′-ATATTTCGAACTTTCATAGAAGGCGGCGGT-3′). Cloning of fluorescent protein and selectable markers into the pEF-myc-cyto-mCMV backbone was performed after digestion with *Nco*I and *Xba*I as well as *Xba*I and *Bst*BI (New England Biolabs, Ipswich, MA, USA).

**Table 2:** Oligonucleotide sequences for molecular biology.

Oligonucleotide	Sequence [5′ → 3′]	Gene
Mock Fw	TCGAGACTAGTCCAGTGA	–
Mock Rev	GATCTCACTGGACTAGTC	–
VEGF-HRE-pair 1 Fw	TCGAGCCACAGTGCATACGTGGGCTCCAACAGGTCCTCTT	VEGF
VEGF-HRE-pair 1 Rev	CTCGACAAGAGGACCTGTTGGAGCCCACGTATGCACTGTGGC	VEGF
VEGF-HRE-pair 2 Fw	GTCGAGCCACAGTGCATACGTGGGCTCCAACAGGTCCTCTT	VEGF
VEGF-HRE-pair 2 Rev	CTCGACAAGAGGACCTGTTGGAGCCCACGTATGCACTGTGG	VEGF
VEGF-HRE-pair 3 Fw	GTCGAGCCACAGTGCATACGTGGGCTCCAACAGGTCCTCTTGTCGA	VEGF
VEGF-HRE-pair 3 Rev	GATCTCGACAAGAGGACCTGTTGGAGCCCACGTATGCACTGTGG	VEGF
AR-ORE-pair 1 Fw	TCGAGTGGAAAATCACC	AR
AR-ORE-pair 1 Rev	CTCGACGGTGATTTTCCAC	AR
AR-ORE-pair 2 Fw	GTCGAGTGGAAAATCACC	AR
AR-ORE-pair 2 Rev	CTCGACGGTGATTTTCCA	AR
AR-ORE-pair 3 Fw	GTCGAGTGGAAAATCACCGTCGA	AR
AR-ORE-pair 3 Rev	GATCTCGACGGTGATTTTCCA	AR

### RNA isolation

Total RNA was isolated from 3 × 10^6^ cells using the miRNeasy Kit (Qiagen, Hilden, Germany) according to the manufacturer’s protocol. RNA concentration and purity were determined using a NanoDrop™ 1000 Spectrophotometer (Thermo Fisher Scientific, Waltham, MA, USA) by absorbance at 260 nm.

### RT-PCR

After RNA isolation, reverse transcription to cDNA was performed with the High Capacity cDNA Reverse Transcription Kit (Thermo Fisher Scientific, Waltham, MA, USA) according to the manufacturer’s protocol. Quantitative real-time PCR on NFAT5 and GAPDH as a loading control was performed using GreenMasterMix No ROX (Genaxxon Bioscience, Ulm, Germany) and the following primers: NFAT5 Fw (5'-TACCACGGACAACAAAGGCA-3'); NFAT5 Rev (5′-AAGTCGATGCCCTTCAGCTC-3′); GAPDH Fw (5′- GACTCTACCCATGGCAAGTTCA-3′); and GAPDH Rev (5′-TCGCTCCTGGAAGATGGTGATG-3′). A LightCycler® 480 Instrument II (Roche Diganostics, Mannheim, Germany) was used for gene expression analysis.

### Flow cytometry analysis

The mean fluorescence intensity of d2EGFP and FKBP-mTagBFP2 expressed under hypoxic and hyperosmotic conditions, respectively, was measured using MACSQuant Analyzer 10 (Miltenyi Biotec, Bergisch-Gladbach, Germany). D2EGFP was measured using a 488 nm laser and the 525/50 nm filter setting (Channel B1), and FKBP-mTagBFP2 was detected by a 405 nm laser and a 450/50 nm filter setting (Channel V1). Subsequent data analysis was performed with MACSQuantify™ Software.

### Live fluorescence microscopy

For live fluorescence microscopy, CHO cells were cultivated in a 2 L bioreactor, and deprivation conditions were induced as described in the methods section Batch fermentation. Automated and continuous sampling was performed by utilizing a peristaltic pump controlled by a socket timer to sample the cell culture suspension every 1 h 30 min. Fluorescence microscopy was performed by integrating a coated µ-slide with Luer adapters and a 0.2 mm channel height (Ibidi GmbH, Gräfelfing, Germany) into a BZ X 800 fluorescence microscope (Keyence, Neu-Isenburg, Germany). Images were automatically recorded by fluorescence microscopy at 5 fixed points in the µ-slide every 1 h 30 min, 3 min after the peristaltic pump stopped sampling to ensure clear good-quality pictures. Fluorescence intensity was qualitatively analyzed by imaging cytometry using Keyence BZ X 800 Analyzing software.

### Statistical analysis

Two-tailed unpaired t test with Welsh’s correction was applied for statistical analysis using GraphPad Prism 6. Data are presented as the mean ± standard deviation.

## Supplementary Information

Supplementary Figure 1.

Supplementary Legends.
