# Supplementary figures and images for: Exploring synthetic biology for the development of a sensor cell line for automated bioprocess control
Source: Sci Rep. 2022 Feb 10;12:2268. doi: 10.1038/s41598-022-06272-x (PMC8831625; doi:10.1038/s41598-022-06272-x)

## Slide 1
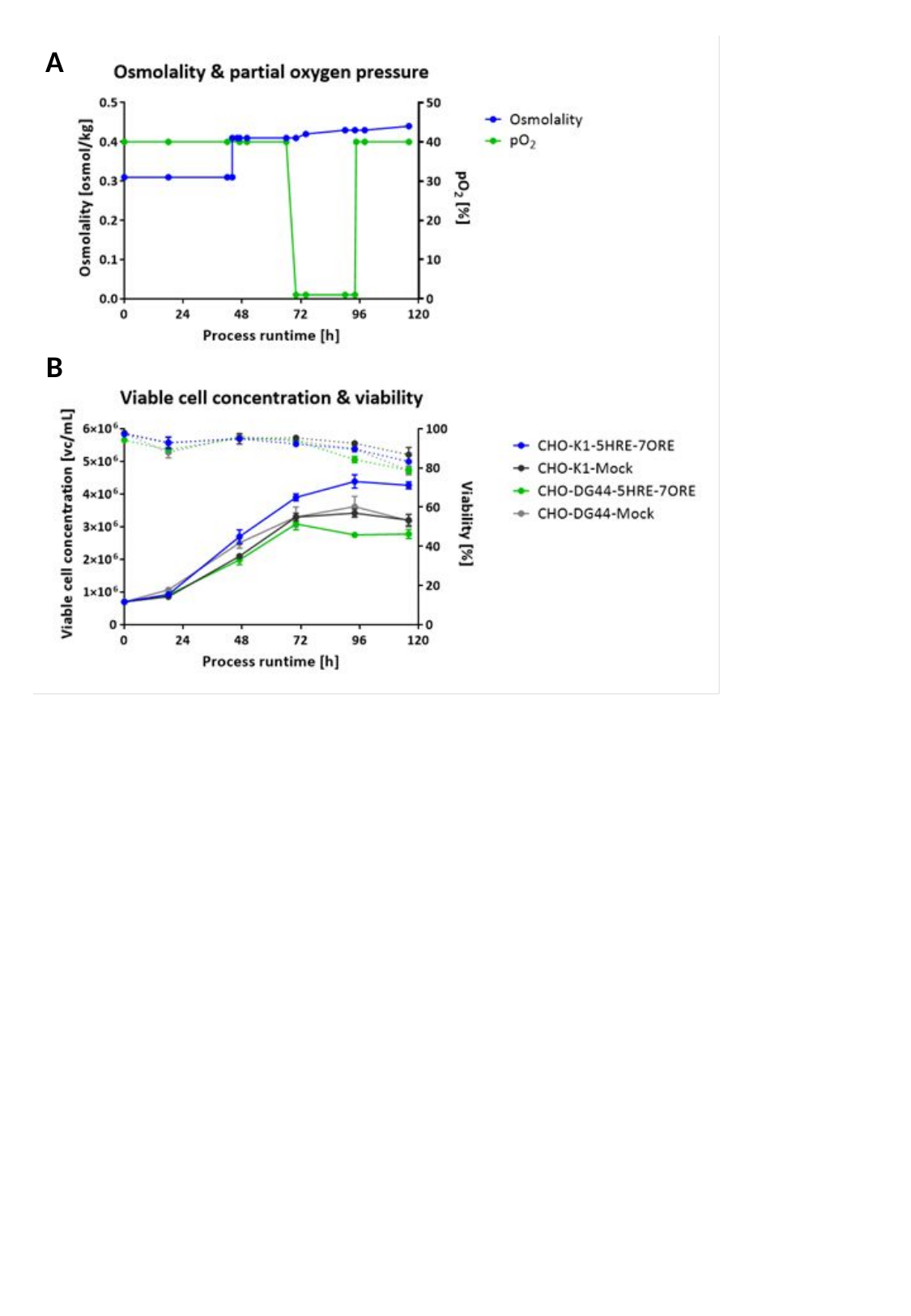

A
B

Supplement: Supplementary file 1 — Supplementary Figure 1. [file 41598_2022_6272_MOESM1_ESM.pptx]
