# Supplementary material for: Exploring synthetic biology for the development of a sensor cell line for automated bioprocess control
Source: Sci Rep. 2022 Feb 10;12:2268. doi: 10.1038/s41598-022-06272-x (PMC8831625; doi:10.1038/s41598-022-06272-x)
Supplement: Supplementary file 2 — Supplementary Legends. [file 41598_2022_6272_MOESM2_ESM.docx]

**Supplemental Figure legends**

**Supplemental Figure 1**: (**A**) Oxygen concentration and osmolality during the batch fermentation. (**B**) Viable cell density and viability of CHO-DG44-5HRE-7ORE, CHO-DG44-Mock, CHO-K1-5HRE-7ORE and CHO-K1-Mock cells during batch fermentation.
